# Supplementary material for: Adding Concomitant Chemotherapy to Postoperative Radiotherapy in Oral Cavity Carcinoma with Minor Risk Factors: Systematic Review of the Literature and Meta-Analysis
Source: Cancers (Basel). 2022 Jul 29;14(15):3704. doi: 10.3390/cancers14153704 (PMC9367295; doi:10.3390/cancers14153704)
Supplement: Supplementary file 1 [file cancers-14-03704-s001.zip › cancers-1781580-supplementary.pdf]

Table S1: Search strings for each database

Database	Search (research performed from 2000 until 23 November 2021)	Number of references
**Pubmed**	(((((ORAL CAVITY CARCINOMA) OR (ORAL CANCER))) OR (ORAL CAVITY TUMOR)) AND (ADJUVANT RADIOCHEMOTHERAPY)) OR (POSTOPERATIVE RADIOCHEMOTHERAPY)<br>(((("mouth"[MeSH Terms] OR "mouth"[All Fields] OR ("oral"[All Fields] AND "cavity"[All Fields]) OR "oral cavity"[All Fields]) AND ("carcinoma"[MeSH Terms] OR "carcinoma"[All Fields] OR "carcinomas"[All Fields] OR "carcinoma s"[All Fields])) OR ("mouth neoplasms"[MeSH Terms] OR ("mouth"[All Fields] AND "neoplasms"[All Fields]) OR "mouth neoplasms"[All Fields] OR ("oral"[All Fields] AND "cancer"[All Fields]) OR "oral cancer"[All Fields]) OR ("mouth neoplasms"[MeSH Terms] OR ("mouth"[All Fields] AND "neoplasms"[All Fields]) OR "mouth neoplasms"[All Fields] OR ("oral"[All Fields] AND "cavity"[All Fields] AND "tumor"[All Fields]) OR "oral cavity tumor"[All Fields])) AND ("chemoradiotherapy, adjuvant"[MeSH Terms] OR ("chemoradiotherapy"[All Fields] AND "adjuvant"[All Fields]) OR "adjuvant chemoradiotherapy"[All Fields] OR ("adjuvant"[All Fields] AND "radiochemotherapy"[All Fields]) OR "adjuvant radiochemotherapy"[All Fields])) OR (("postoperative period"[MeSH Terms] OR ("postoperative"[All Fields] AND "period"[All Fields]) OR "postoperative period"[All Fields] OR "postop"[All Fields] OR "postoperative"[All Fields] OR "postoperatively"[All Fields] OR "postoperatives"[All Fields]) AND ("chemoradiotherapy"[MeSH Terms] OR "chemoradiotherapy"[All Fields] OR "radiochemotherapy"[All Fields]))	8049
**Embase**	('**mouth carcinoma**' OR '**mouth cancer**' OR '**mouth tumor**') AND '**adjuvant chemoradiotherapy**' OR '**postoperative chemoradiotherapy**' OR '**adjuvant chemoradiation**' OR '**postoperative chemoradiation**'	2579
**Cochrane Library**	Search Name: oral carcinoma in Title Abstract Keyword AND chemoradiotherapy in Title Abstract Keyword (Word variations have been searched)<br><br>ID Search<br>#1 oral carcinoma:ti,ab,kw AND chemoradiotherapy:ti,ab,kw with Cochrane Library publication date Between Jan 2000 and Dec 2021 (Word variations have been searched)	413 references trials; 6 cochrane reviews

Table S2: Excluded full papers (numbered on the basis of the References) with main reason

9.	Yanamoto S.	Multicenter Retrospective Study of Adjuvant Therapy for Patients with Pathologically Lymph Node-Positive Oral Squamous Cell Carcinoma: Analysis of Covariance Using Propensity Score. Ann Surg Oncol, 2015. 22 Suppl 3: p. S992-9.	No separated data for major and minor risk factors
10.	Takahashi (2014)	Effects of postoperative chemotherapy and radiotherapy on patients with squamous cell carcinoma of the oral cavity and multiple regional lymph node metastases. Int J Oral Maxillofac Surg. 2014 Jun;43(6):680-5. doi: 10.1016/j.ijom.2013.11.013. Epub 2013 Dec 30. PMID: 24387949.	Only major risk factors
11.	De Almeida (2020)	Treatment implications of postoperative chemoradiotherapy for squamous cell carcinoma of the oral cavity with minor and major extranodal extension https://doi.org/10.1016/j.oraloncology.2020.104845	Only major risk factors
12.	Fan K.H.	Combined-modality treatment for advanced oral tongue squamous cell carcinoma. Int J Radiat Oncol Biol Phys, 2007. 67(2): p. 453-61.	Population analyzed in Fan K.H., 2014
13.	Yamada (2021)	A retrospective investigation of minor risk factors as prognostic predictors and treatment indications in oral squamous cell carcinoma J Dent Sci. 2021 Jan;16(1):445-452. doi: 10.1016/j.jds.2020.09.012. Epub 2020 Oct 7. PMID: 33384833; PMCID: PMC7770354.	Only major risk factors
14.	Lee L-Y, Lin C-Y, Cheng N-M, et al.	Poor tumor differentiation is an independent adverse prognostic variable in patients with locally advanced oral cavity cancer— Comparison with pathological risk factors according to the NCCN guidelines. Cancer Med. 2021;10:6627– 6641.	Only major risk factors
15.	Kim TH, Cha I-H, Choi EC, Kim HR	Postoperative Concurrent Chemoradiotherapy Versus Radiotherapy Alone for Advanced Oral Cavity Cancer in the Era of Modern Radiation Techniques. Front. Oncol 2021. 11:619372.	No separated data for major and minor risk factors
16.	Anand, A.K., et al.	Significance of perineural invasion in locally advanced bucco alveolar complex carcinomas treated with surgery and postoperative radiation ± concurrent chemotherapy. Head Neck, 2017. 39(7): p. 1446-1453	No separated data for major and minor risk factors
17.	Chen CC 2017	Comparison cisplatin with cisplatin plus 5FU in head and neck cancer patients received postoperative chemoradiotherapy. Oral Oncol, 2017. 69: p. 11-14	Only major risk factors
18.	Hasegawa, T., et al.	Multi-center retrospective study of the prognosis and treatment outcomes of Japanese oral squamous cell carcinoma patients with single lymph node metastasis and extra nodal extension. J Surg Oncol, 2018. **117**(8): p. 1736-1743	No separated data for major and minor risk factors
19.	Hasegawa, T., et al	Multicenter retrospective study of the prognosis and treatment outcomes of Japanese oral squamous cell carcinoma patients with level IV/V metastasis. Head Neck, 2019. 41(7): p. 2256-2263	No separated data for major and minor risk factors
20.	Brooker, R.C., et al.,	Adjuvant management of locally advanced oral squamous cell carcinoma - real-world challenges and opportunities. Br J Oral Maxillofac Surg, 2021. 59(8): p. 952-958.	Registry study
21.	Chan, A.K., et al.,	Postoperative intensity-modulated radiotherapy following surgery for oral cavity squamous cell carcinoma: patterns of failure. Oral Oncol, 2013. 49(3): p. 255-60.	No separated data for major and minor risk factors
22.	Harari, P.M., et al.,	Postoperative chemoradiotherapy and cetuximab for high-risk squamous cell carcinoma of the head and neck: Radiation Therapy Oncology Group RTOG-0234. Journal of Clinical Oncology, 2014. 32(23): p. 2486-2495.	No separated data for major and minor risk factors
23.	Hingsammer, L., et al.	The influence of lymph node ratio on survival and disease recurrence in squamous cell carcinoma of the tongue. Int J Oral Maxillofac Surg, 2019. 48(7): p. 851-856	No separated data for major and minor risk factors

24.	Hinerman, R.W., et al.,	Postoperative irradiation for squamous cell carcinoma of the oral cavity: 35-year experience. Head Neck, 2004. 26(11): p. 984-94	No Adjuvant CRT
25.	Hasegawa, T., et al.	Retrospective study of treatment outcomes after postoperative chemoradiotherapy in Japanese oral squamous cell carcinoma patients with risk factors of recurrence. Oral Surg Oral Med Oral Pathol Oral Radiol, 2017. 123(5): p. 524-530	No separated data for major and minor risk factors
26.	Jardim, J.F., et al.	Prognostic impact of perineural invasion and lymphovascular invasion in advanced stage oral squamous cell carcinoma. Int J Oral Maxillofac Surg, 2015. 44(1): p. 23-8	Only major risk factors
27.	Kademani, D., et al.	Prognostic factors in intraoral squamous cell carcinoma: the influence of histologic grade. J Oral Maxillofac Surg, 2005. 63(11): p. 1599-605.	No adjuvant CRT
28.	Kang, C.J., et al.,	The number of pathologically positive lymph nodes and pathological tumor depth predicts prognosis in patients with poorly differentiated squamous cell carcinoma of the oral cavity. Int J Radiat Oncol Biol Phys, 2011. 81(4): p. e223-30	No separated data for major and minor risk factors
29.	Kreppel, M., et al.,	Combined-modality treatment in advanced oral squamous cell carcinoma: Primary surgery followed by adjuvant concomitant radiochemotherapy. Strahlenther Onkol, 2011. 187(9): p. 555-60.	CRT for major risk factors
30.	Lee, J.H., et al.,	Adjuvant postoperative radiotherapy with or without chemotherapy for locally advanced squamous cell carcinoma of the head and neck: The importance of patient selection for the postoperative chemoradiotherapy. Cancer Research and Treatment, 2013. 45(1): p. 31-39	No separated data for site and major and minor risk factors
31.	Liao, C.T., et al.,	Surgical outcome of T4a and resected T4b oral cavity cancer. Cancer, 2006. 107(2): p. 337-44	POCRT done also in presence of ECE
32.	Liao, C.T., et al.,	Neck dissection field and lymph node density predict prognosis in patients with oral cavity cancer and pathological node metastases treated with adjuvant therapy. Oral Oncology, 2012. 48(4): p. 329-336.	POCRT done also in presence of major RF
33.	Liao, C.T., et al.,	Pathological risk factors stratification in pN3b oral cavity squamous cell carcinoma: Focus on the number of positive nodes and extranodal extension. Oral Oncol, 2018. 86: p. 188-194	POCRT done also in presence of major RF
34.	Lin, Y.W., et al.,	Patterns of failure after postoperative intensity-modulated radiotherapy for locally advanced buccal cancer: Initial masticator space involvement is the key factor of recurrence. Head Neck, 2018. 40(12): p. 2621-2632	POCRT done only for major risk factors
35.	Maihoefer, C., et al.,	Postoperative (chemo) radiation in patients with squamous cell cancers of the head and neck - clinical results from the cohort of the clinical cooperation group "Personalized Radiotherapy in Head and Neck Cancer". Radiat Oncol, 2018. 13(1): p. 123	No separated data for site and major and minor risk factors
36.	Mair, M.D., et al.,	Depth of invasion, size and number of metastatic nodes predicts extracapsular spread in early oral cancers with occult metastases. Oral Oncology, 2018. 81: p. 95-99	No data minor risk factors
37.	Lee, A., et al.,	Utilization and Survival of Postoperative Radiation or Chemoradiation for pT1-2N1M0 Head and Neck Cancer. Otolaryngol Head Neck Surg, 2018. 158(4): p. 677-684.	Mixed population with less than 40 percent of OCC
38.	Lee, Y.G., et al.,	Treatment strategy and outcomes in locally advanced head and neck squamous cell carcinoma: a nationwide retrospective cohort study (KCSG HN13-01). BMC Cancer, 2020. 20(1): p. 813.	Missing data about adjuvant RTCT
39.	Leeman, J.E., et al.,	Patterns of Treatment Failure and Postrecurrence Outcomes Among Patients With Locally Advanced Head and Neck Squamous Cell Carcinoma After Chemoradiotherapy Using Modern Radiation Techniques. JAMA Oncol, 2017. 3(11): p. 1487-1494.	POCRT performed mainly for major RF
40.	Lin, C.S., et al.,	Squamous cell carcinoma of the buccal mucosa: an aggressive cancer requiring multimodality treatment. Head Neck, 2006. 28(2): p. 150-7.	Not POCRT, only PORT
41.	Liu, T., et al.,	The utility of postoperative radiotherapy in intermediate-risk oral squamous cell carcinoma. Int J Oral Maxillofac Surg, 2021. 50(2): p. 143-150.	PORT, not POCRT

42.	Lo, W.L., et al.,	Outcomes of oral squamous cell carcinoma in Taiwan after surgical therapy: factors affecting survival. J Oral Maxillofac Surg, 2003. 61(7): p. 751-8.	Not related to the topic
43.	Luukkaa, M., et al.,	Treatment of squamous cell carcinoma of the oral cavity, oropharynx and hypopharynx--an analysis of 174 patients in south western Finland. Acta Oncol, 2003. 42(7): p. 756-62.	Patients treated with POCRT not specified in the study
44.	Ma'aita, J.K.,	Oral cancer in Jordan: a retrospective study of 118 patients. Croat Med J, 2000. 41(1): p. 64-9.	Patients treated with POCRT not specified in the study
45.	Mair, M.D., et al.,	Impact of radical treatments on survival in locally advanced T4a and T4b buccal mucosa cancers: Selected surgically treated T4b cancers have similar control rates as T4a. Oral Oncol, 2018. 82: p. 17-22.	POCRT done in presence of major risk factors
46.	Marra, A., et al.,	Long-term disease-free survival in surgically-resected oral tongue cancer: a 10-year retrospective study. Acta Otorhinolaryngol Ital, 2019. 39(2): p. 84-91.	POCRT done in presence of major risk factors
47.	Martínez Carrillo, M., et al.,	Selective use of postoperative neck radiotherapy in oral cavity and oropharynx cancer: a prospective clinical study. Radiat Oncol, 2013. 8: p. 103.	POCRT done in presence of major risk factors
48.	McMahon, J.D., et al.,	Oral and oropharyngeal cancer in the West of Scotland-long-term outcome data of a prospective audit 1999-2001. Br J Oral Maxillofac Surg, 2011. 49(2): p. 92-8.	Risk factors considered for POCRT not specified in the study
49.	Metcalfe, E., et al.,	Postoperative (Chemo)Radiotherapy for Oral Cavity Squamous Cell Carcinomas: Outcomes and Patterns of Failure. Clin Oncol (R Coll Radiol), 2017. 29(1): p. 51-59.	POCRT done in presence of major risk factors
50.	Mroueh, R., et al.,	Improved outcomes with oral tongue squamous cell carcinoma in Finland. Head Neck, 2017. 39(7): p. 1306-1312.	POCRT done in presence of major risk factors
51.	Murthy, V., et al.,	Analysis of prognostic factors in 1180 patients with oral cavity primary cancer treated with definitive or adjuvant radiotherapy. J Cancer Res Ther, 2010. 6(3): p. 282-9.	POCRT done mainly in presence of major risk factors
52.	Nair, D., et al.,	Perineural invasion: Independent prognostic factor in oral cancer that warrants adjuvant treatment. Head Neck, 2018. 40(8): p. 1780-1787.	POCRT done mainly in presence of major risk factors
53.	Newman, M., et al.,	Relationship of depth of invasion to survival outcomes and patterns of recurrence for T3 oral tongue squamous cell carcinoma. Oral Oncol, 2021. 116: p. 105195.	POCRT done in presence of major risk factors
54.	Noble, A.R., et al.,	Risk Factors Associated with Disease Recurrence in Patients with Stage III/IV Squamous Cell Carcinoma of the Oral Cavity Treated with Surgery and Postoperative Radiotherapy. Anticancer Res, 2016. 36(2): p. 785-92.	POCRT done in presence of major risk factors
55.	Osman, N., et al.,	Weekly cisplatin concurrently with radiotherapy in head and neck squamous cell cancer: a retrospective analysis of a tertiary institute experience. Eur Arch Otorhinolaryngol, 2014. 271(8): p. 2253-9.	Mixed population with definitive RTCT and POCRT with not well specified criteria
56.	Palazzi, M., et al.,	Patterns of postoperative radiotherapy for head and neck cancer in Italy: a prospective, observational study by the head and neck group of the Italian Association for Radiation Oncology (AIRO). Tumori, 2011. 97(2): p. 170-6.	POCRT done mainly in presence of major RF
57.	Pillai, V., et al.,	Prognostic determinants of locally advanced buccal mucosa cancer: Do we need to relook the current staging criteria? Oral Oncol, 2019. 95: p. 43-51.	POCRT done also in presence of major RF

58.	Quinsan, I.C.M., et al.,	Functional outcomes and survival of patients with oral and oropharyngeal cancer after total glossectomy. Braz J Otorhinolaryngol, 2020. 86(5): p. 545-551.	Mixed population with radical RTCT and surgery
59.	Rades, D., et al.,	Cisplatin superior to carboplatin in adjuvant radiochemotherapy for locally advanced cancers of the oropharynx and oral cavity. Strahlenther Onkol, 2012. 188(1): p. 42-8.	POCRT done also in presence of major RF
60.	Santos, F.M.D., G.A. Viani, and J.F. Pavoni,	Evaluation of survival of patients with locally advanced head and neck cancer treated in a single center. Braz J Otorhinolaryngol, 2021. 87(1): p. 3-10.	POCRT done in presence of major RF
61.	Jensen, J.S., et al.,	The Copenhagen Oral Cavity Squamous Cell Carcinoma database: protocol and report on establishing a comprehensive oral cavity cancer database. Clin Epidemiol, 2019. 11: p. 733-741.	Criteria for POCRT not specified in the article
62.	Sher, D.J., et al.,	Treatment of oral cavity squamous cell carcinoma with adjuvant or definitive intensity-modulated radiation therapy. Int J Radiat Oncol Biol Phys, 2011. 81(4): p. e215-22.	POCRT done in presence of major RF
63.	Shia, B.C., et al.,	Outcomes for Elderly Patients Aged 70 to 80 Years or Older with Locally Advanced Oral Cavity Squamous Cell Carcinoma: A Propensity Score-Matched, Nationwide, Oldest Old Patient-Based Cohort Study. Cancers (Basel), 2020. 12(2).	No clear data about adjuvant RTCT
64.	Silva, P.B., et al.,	Prognostic factors on surgically and non-surgically treated oral squamous cell carcinoma: Advances in survival in fifteen years of follow up. J Clin Exp Dent, 2021. 13(3): p. e240-e249.	No data about POCRT
65.	Spiotto, M.T., et al.,	Differences in Survival With Surgery and Postoperative Radiotherapy Compared With Definitive Chemoradiotherapy for Oral Cavity Cancer: A National Cancer Database Analysis. JAMA Otolaryngol Head Neck Surg, 2017. 143(7): p. 691-699.	No clear data about POCRT
66.	Suzuki, M., et al.,	Phase I study of weekly docetaxel infusion and concurrent radiation therapy for head and neck cancer. Jpn J Clin Oncol, 2003. 33(6): p. 297-301.	Data about POCRT in OCC not clear
67.	Tangthongkum, M., et al.,	Treatment outcome of advance staged oral cavity cancer: concurrent chemoradiotherapy compared with primary surgery. Eur Arch Otorhinolaryngol, 2017. 274(6): p. 2567-2572.	POCRT in presence of major RF
68.	Thakar, A., et al.	Laryngoscope, 2021. 131(10): p. 2254-2261. Oral Cancer in the Indian Subcontinent-Survival Outcomes and Risk Factors with Primary Surgery	POCRT in presence of major RF
69.	Wan, X.C., A.M. Egloff, and J. Johnson	Histological assessment of cervical lymph node identifies patients with head and neck squamous cell carcinoma (HNSCC): Who would benefit from chemoradiation after surgery? Laryngoscope, 2012. 122(12): p. 2712-2722.	Mixed population and more than half of patients with ECE
70.	Yokota, T., et al.	Prognostic Factors and Multidisciplinary Postoperative Chemoradiotherapy for Clinical T4a Tongue Cancer. Oncology, 2016. 91(2): p. 78-84.	POCRT done mainly in patients with major risk factors
71.	Zanoni, D.K., et al.	Survival outcomes after treatment of cancer of the oral cavity (1985–2015). Oral Oncology, 2019. 90: p. 115-121.	Not possible extrapolation of data for OCC
72.	Zumsteg, ZS, et al	Quantitative lymph node burden as a 'very-high-risk' factor identifying head and neck cancer patients benefiting from postoperative chemoradiation. Ann Oncol. 2019 Oct 1;30(10):1669. doi: 10.1093/annonc/mdz172. Erratum for: Ann Oncol. 2019 Jan 1;30(1):76-84. PMID: 31168574; PMCID: PMC6857612.	POCRT done also in presence of major RF; outcome of minor RF not evaluable
